# Preparation of poly-l-lysine-based nanoparticles with pH-sensitive release of curcumin for targeted imaging and therapy of liver cancer *in vitro* and *in vivo*

**DOI:** 10.1080/10717544.2018.1461957

**Published:** 2018-04-16

**Authors:** Dae Hyeok Yang, Hyun Joo Kim, Kyeongsoon Park, Jae Kwang Kim, Heung Jae Chun

**Affiliations:** a Institute of Cell and Tissue Engineering, College of Medicine, The Catholic University of Korea, Seoul, Republic of Korea;; b Department of Biomedical Sciences, College of Medicine, The Catholic University of Korea, Seoul, Republic of Korea;; c Department of Systems Biotechnology, Chung-Ang University, Anseong, Republic of Korea;; d Department of Internal Medicine, Bucheon St. Mary’s Hospital, College of Medicine, The Catholic University of Korea, Bucheon, Republic of Korea

**Keywords:** Poly-L-lysine nanoparticles, curcumin, PEGylation, cyanine 5.5, theranosis

## Abstract

Poly-l-lysine (PLL) nanoparticle (NP) system was prepared for the controlled release of curcumin (CUR) by pH stimuli, and its theranostic efficacy on cancer was compared to that of CUR solution *in vitro* and *in vivo*. Deoxycholic acid (DOCA), methoxy polyethylene glycol (MPEG) and cyanine 5.5 (cy5.5) were conjugated to the amine group of PLL through condensation reaction (PLL-DOCA-MPEG-cy5.5), followed by encapsulation of CUR by dialysis method (PLL-DOCA-MPEG-cy5.5/CUR NPs). The composition, morphology and size distribution of PLL-DOCA-MPEG-cy5.5 NPs were characterized by proton nuclear magnetic resonance (^1^H NMR), transmission electron microscopy (TEM) and dynamic light scattering (DLS), respectively. *In vitro* tests exhibited that changes in the charge and size of the NPs at low pH led to the improved cellular uptake of CUR into human hepatoma Hep3B cell line by electrostatically absorptive endocytosis. PEGylation with MPEG was turn out to be very effective to have a prolonged blood circulation time, in turn increased the EPR effect. In addition, the incorporation of Cy5.5 into NPs provided successful biodistribution images *in vivo* and *ex vivo*. Our findings suggest that PLL-DOCA-MPEG-cy5.5/CUR NPs may have promising applications in cancer theranosis.

## Introduction

Curcumin (CUR) is a natural polyphenol extracted from turmeric and has received considerable attention in recent decades because of its potential utility in the prevention and therapeutic treatment of a variety of cancers (Anand et al., 2007; Wang et al., 2017). Recent investigations have also shown that CUR may improve anti-tumor activity through a reversal effect on multi-drug resistance (MDR), a major obstacle of conventional chemotherapy; therefore, it is in clinical trials for several types of cancer (Anand et al., 2007; Dhillon et al., 2008; Lv et al., 2016). However, the poor water solubility and low systemic bioavailability of CUR still limit the clinical use (Anand et al., 2007).

To address these problems, drug delivery systems (DDSs) using different carriers such as liposomes, phospholipids and nanoparticles have been extensively employed (Peng & Qian, 2014). Among the candidate carriers, polymeric nanoparticles (NPs) may have various functions including targeting and imaging against cancer cells by conjugation of multiple specific ligands and contrast agents, respectively (Sinha et al., 2006). Polymeric NPs can encapsulate anticancer drugs into their cores, thereby improving the drug solubility and delivery efficiency to the target sites (Narvekar et al., 2014). Also, polymeric NPs can be designed in accordance with the proper shape and size for the controlled release of therapeutic agents into cancer cells (Wang et al., 2014).

As a material that meets the advantages of polymeric NPs, poly-l-lysine (PLL) has gained an attraction in cancer theranostic applications. Hydrophobic substances with carboxyl groups can be chemically conjugated to the amine groups of PLL, thus encapsulating lipophilic drugs during NP formation (Zhou et al., 2015). In addition, it becomes possible to target cancer and conduct diagnosis by binding ligands and contrast agents with the amine groups (Zhou et al., 2015). In addition to the merits of conjugation, another important aspect of the amine group of PLL is its conversion capability into a positively charged hydrophilic amino group under acidic conditions, leading to an electrostatic interaction with the negatively-charged cancer cell membranes (Vasir & Labhasetwar, 2008). PLL, as a polycationic peptide, can be selectively internalized into cancer cells via electrostatically adsorptive endocytosis (Vasir & Labhasetwar, 2008).

**Figure 1.**
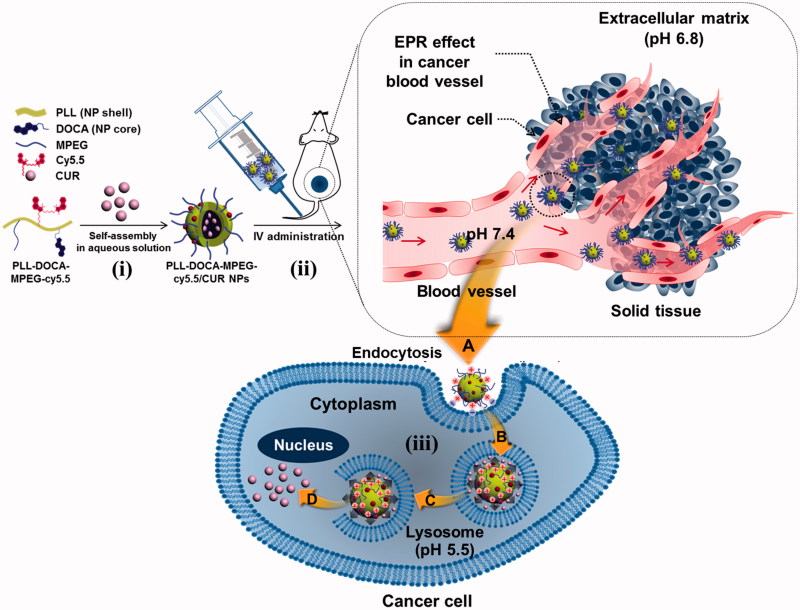
Schematic illustration of the endocytosis of PLL-DOCA-MPEG-cy5.5/CUR NPs into cancer cells and mechanism of the NPs on the apoptosis of the cells.

In the present study, we prepared NPs based on PLL as theranostic agents for CUR delivery to cancer cells (Figure 1). Deoxycholic acid (DOCA) was conjugated with PLL as a core for enhanced CUR encapsulation through hydrophobic interaction (PLL-DOCA). NPs were then PEGylated with methoxy polyethylene glycol (MPEG) for stealth effect and coupled with cyanine 5.5 (cy5.5) for fluorescence imaging (PLL-DOCA-MPEG-cy5.5 NPs). The theranostic capacity of the CUR-loaded NPs (PLL-DOCA-MPEG-cy5.5/CUR NPs) was investigated using the human hepatoma Hep3B cell line *in vitro* and using a cancer-bearing mouse model *in vivo*.

## Materials and methods

### Materials

Poly-l-lysine⋅hydrobromide (PLL⋅HBr, Mw: 1–5 kDa), deoxycholic acid (DOCA), methoxy polyethylene glycol acetic acid (MPEG-COOH, Mw: 5K), CUR, pyrene, deuterium oxide (D_2_O), dimethyl solfoxide-*_d6_* (DMSO-*_d6_*), and phosphate-buffered saline (PBS, pH 7.4) were purchased from Sigma-Aldrich (St. Louis, MO). Cyanine 5.5 carboxylic acid (cy5.5-COOH) was obtained from Lumiprobe Corporation (Hallandale Beach, FL). 4-(4,6-Dimethoxy-1,3,5-triazin-2-yl)-4-methylmorpholinium chloride (DMT-MM) was supplied from Wako Pure Chemical Industries (Osaka, Japan). Dialysis tubes (Spectrum Laboratories Inc., Rancho Dominguez, CA) were used for purification. Acidic PBS (pH 5.5 and 6.8) was adjusted using hydrochloric acid (HCl). The human hepatoma Hep3B cell line was supplied by the Korean Cell Line Bank (Seoul, Republic of Korea). High glucose Dulbecco’s modified eagle medium (DMEM), fetal bovine serum (FBS), and penicillin–streptomycin (PS) were used for cell culture. Cell counting kit-8 (CCK-8) was purchased from Dojindo Molecular Technologies, Inc. (Kumamoto, Japan). The nuclei of the cells were counterstained with ProLong^®^ Gold anti-fade reagent with 4′,6-diamidino-2-phenylindole (DAPI, Invitrogen, OR). The AP *In Situ* Cell Death Detection Kit was purchased from Roche Life Science (Indianapolis, IN). All chemicals were used as received without further purification.

### Preparation and characterization of DOCA/MPEG/cy5.5-conjugated PLL (PLL-DOCA-MPEG-cy5.5)

**Figure 2.**
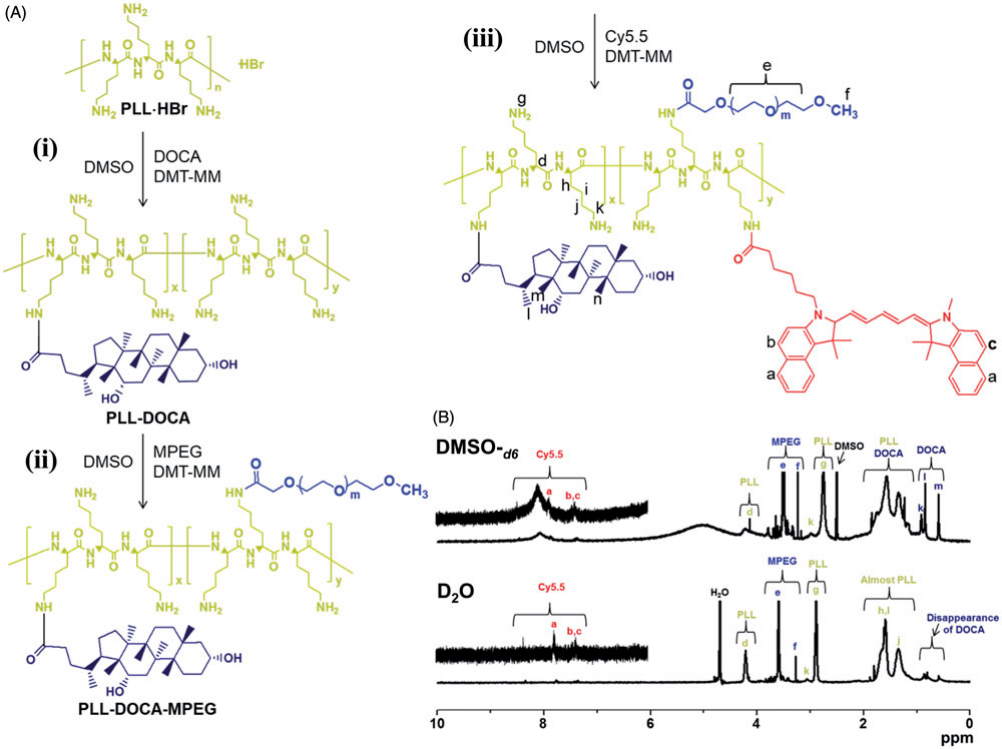
Schematic illustration of the preparation of PLL-DOCA-MPEG-cy5.5, and (B) ^1^H NMR spectra of PLL-DOCA-MPEG-cy5.5 examined using DMSO-*_d6_* and D_2_O.

DOCA, MPEG, and cy5.5 to PLL were conjugated to PLL through condensation reaction (Figure 2(A)) (Zhou et al., 2015). Briefly, (i) the DOCA (0.02 mmol, 7.5 mg) and DMT-MM (0.04 mmol, 10 mg) were dissolved in DMSO (30 mL), and stirred at room temperature for 1 h. The mixture was added to an aqueous PLL⋅HBr (0.4 μmol, 30 mg) solution and was reacted at room temperature for 2 days. The resultant solution was dialyzed in a dialysis tube (cut-off 20 kDa) against a co-solvent of ethanol and water (50/50 v/v%) at room temperature. After 3 days, the purified solution was lyophilized at –90 °C for 7 days (PLL-DOCA). According to the preparation method of PLL-DOCA, (ii and iii) MPEG-COOH (0.001 mmol, 1.0 mg) and cy5.5-COOH (0.001 μmol, 1.0 mg) were conjugated to PLL-DOCA (0.4 μmol, 30 mg). The resultant products were designated as PLL-DOCA-MPEG and PLL-DOCA-MPEG-cy5.5, respectively.

### Determination of critical micelle concentration (CMC) of PLL-DOCA-MPEG-cy5.5

The CMC was determined by fluorescence spectroscopy of pyrene in aqueous solution (Wilhelm et al., 1991). Pyrene was used as a probe molecule. Pyrene solution (0.1 mg/L in acetone) was adjusted to 1.2 × 10^−6 ^ M in water and acetone was evaporated at 40 °C. After 2 hours, the pyrene solution was mixed with various concentrations of PLL-DOCA-MPEG-cy5.5 (6.1 × 10^−5^ – 1.0 mg/mL) in DMSO (6.0 × 10^−7 ^ M), and then dialyzed against water in a dialysis tube (cut-off 100–500 Da) at room temperature for 3 days. CMC was calculated as the intensity ratio of the two emission spectra of pyrene (*I*_338_/*I*_332_).

### *In vitro* cytotoxicity assay of PLL-DOCA-MPEG-cy5.5 NPs

The Hep3B cell line was sub-cultured for *in vitro* and *in vivo* assays. The cell line was cultured with high glucose DMEM supplemented with 10% FBS and 1% PS in an incubator at 37 °C in humid air with 5% CO_2_. The media was changed every 3 days during culture. The cells were passaged at 80% confluence. The cytotoxicity of PLL-DOCA-MPEG-cy5.5 NPs on Hep3B cells (ATCC^®^, HB-8064™) was assessed using a CCK-8 reagent. The cells (5 × 10^3^ cells/well) were incubated with various NP concentrations (0, 3.125, 6.25, 12.5, 25, 50, and 100 μg/mL culture media) for 24, 48, and 72 hours. At the predetermined time intervals, each sample was washed three times with PBS (pH 7.4) and exchanged with fresh medium. The CCK-8 reagent (10 μL) was added to the samples and incubated for an additional 4 hours. The optical density was measured by a microplate reader at 450 nm.

### Preparation and characterization of CUR-loaded NPs (PLL-DOCA-MPEG-cy5.5/CUR NPs)

PLL-DOCA-MPEG-cy5.5/CUR NPs were prepared using a dialysis method (Nguyen et al., 2015). Methanolic CUR (1 mg/5 mL in methanol) was dropped into a solution of PLL-DOCA-MPEG-cy5.5 in DMSO (30 mL). The solution was dialyzed in a dialysis tube (cut-off 1 kD) against water at room temperature for 3 days. After centrifugation, the supernatant was extracted and lyophilized at −90 °C for 7 days. The morphology of the CUR-loaded NPs was observed by transmission electron microscopy (TEM, JEOL Ltd., Tokyo, Japan). The size distribution, polydispersity index (PDI), and zeta potential of PLL-DOCA-MPEG-cy5.5/CUR NPs on pH change (pH 5.5, 6.8, and 7.4) were characterized by dynamic light scattering (DLS, Nano ZS Zetasizer, Malvern Instruments Ltd., UK) measurement. The encapsulation efficiency (EE) and loading capacity (LC) of CUR were calculated by analyzing the amount of removed CUR, as characterized by ultraviolet-visible (UV–Vis, UV-1650 PC, Shimadzu, Japan) spectroscopy at 420 nm. A stock solution of CUR was diluted to a range of 5–50 μmol/L. The standard calibration curve of CUR was obtained by plotting the different concentrations. EE and LC were calculated according to the following equations (Tiwari et al., 2017).

(1) $$\text{EE}\;\text{(\%)}\text{=}\frac{\text{Wt-Wf}}{\text{Wt}}\text{×100}$$

(2) $$\text{LC}\;\text{(\%)}\text{=}\frac{\text{Wt-Wf}}{\text{Wn}}\text{×100}$$

Wt, Wf, and Wn represent the total amount of CUR, amount of free CUR, and amount of freeze-dried PLL-DOCA-MPEG-cy5.5/CUR NPs, respectively. The resultant solution was lyophilized at −90 °C for 7 days and stored in a refrigerator (−20 °C) for further use.

### *In vitro* anti-tumor activity of PLL-DOCA-MPEG-cy5.5/CUR NPs

CCK-8 reagent was used to investigate the anti-tumor activity of PLL-DOCAMPEG/cy5.5/CUR NPs (corresponding to 0.09 μM CUR) on Hep3B cells, compared with control and free CUR solution (corresponding to 0.09 μM CUR) (Xu et al., 2014). Various concentrations of the NP were added to the cells (5 × 10^3^ cells/well) and incubated for 24, 48, and 72 hours. After washing the cells at each of the incubation time, they were treated with the reagent, followed by incubation for an additional 4 hours. The optical density was measured by a microplate reader at 450 nm.

### Release test of CUR

Aliquots of PLL-DOCA-MPEG-cy5.5/CUR NP dispersions (21.5 mg/1 mL) were loaded into a dialysis tube (cut-off 20 kDa). Each dialysis tube was immersed into three PBS solutions (10 mL; pH 5.5, 6.8, and 7.4) and agitated at 37 °C with 100 rpm. The three pH values (5.5, 6.8, and 7.4) were used to simulate different biological environments: endosomes/lysosome, cancer microenvironment, and normal physiological condition. At predetermined time intervals (1, 2, 4, 6, 8, 12, 24, 36, 48, 60, and 72 hours), the released media was collected, and the same volume of fresh media was replenished. The amount of released CUR was determined by ultraviolet–visible (UV–Vis) spectroscopy at 420 nm (Yang, et al., 2017).

### *In vitro* cellular uptake assay of CUR

The *in vitro* cellular uptake of CUR in CUR solution and PLL-DOCA-MPEG-cy5.5/CUR NPs was examined by confocal laser scanning microscopy (CLSM, LSM 510 Meta, Zeiss, Oberkochen, Germany) and flow cytometry (FACS Canto II, BD Biosciences, San Jose, CA). Hep3B cells stained with DAPI were used as a control. Hep3B cells (1 × 10^5^ cells/mL) were seeded on PLL-coated glass coverslips and incubated with the media overnight in an incubator at 37 °C in humid air with 5% CO_2_. The cells were treated with CUR solution (3 mL, pH 7.4 PBS) or PLL-DOCA-MPEG-cy5.5/CUR NP solution (3 mL), each in 50 mL of medium (corresponding to 0.09 μM CUR) for 5 and 10 minutes, and then washed with PBS (pH 7.4) three times. The solution-treated cells were fixed in 4% (v/v) formaldehyde solution for 10 minutes and washed with PBS (pH 7.4) three times. The fixed cells were counter-stained with 10 μL ProLong^®^ Gold antifade reagent with DAPI, as visualized by CLSM at an excitation of 488 nm. For flow cytometry assay, untreated Hep3B cells were used as a control. The analysis was performed using an annexin V-cy3 detection kit (Abcam^®^, Cambridge, MA) according to the manufacturer’s instructions. The cells treated with the samples for 5 and 10 minutes (corresponding to 0.09 μM CUR) were washed three times with PBS (pH 7.4) and incubated overnight. The cells were harvested with 0.25% trypsin/EDTA, and then transferred to 5 mL tubes. After addition of 5 μL of annexin V-cy3 and 10 μg/mL of DAPI, the samples were gently mixed and incubated for 30 minutes in the absence of light. Then, 10,000 cells were acquired for flow cytometric analysis.

### Establishment of a Hep3B cancer-bearing mouse model

BALB/c nude mice (*n* = 35, 22–25 g, D. Y. Biotech., Seoul, Korea) were used for the evaluation of *in vivo* NIRF imaging, *ex vivo* biodistribution, and *in vivo* anti-tumor effect. For the establishment of a Hep3B cancer-bearing mouse model, Hep3B cells (5 × 10^6^) suspended in 100 μL of water (JEIL PHARMACEUTICAL Co. Ltd., Daegu, Korea) were administered subcutaneously into the backs of the mice. The mice were maintained in a light-controlled room at 20 °C with a relative humidity of 50–60%. The evaluation of imaging and anti-tumor effect was performed on mice with cancer volumes of 150–200 mm^3^ and 250–330 mm^3^, respectively.

### Evaluation of *in vivo* NIRF imaging and *ex vivo* biodistribution

PLL-DOCA-MPEG-cy5.5/CUR NPs (5 mg/kg in 100 μL of water for injection; 189 μg/kg of CUR) were administered via the lateral tail vein of Hep3B cancer-bearing mice (*n* = 5), and the fluorescence intensities were monitored by a real-time IVIS imaging system 200 (Xenogen Corporation, CA) over time. The NIRF imaging was performed on mice with a tumor volume of 150–200 mm^3^. Laser diodes with wavelengths of 670 nm and 480 nm were used for the excitation of cy5.5 and CUR, respectively. The fluorescence intensities were scanned at 1, 3, 6, 12, 24, 38, 72, and 120 hours. To confirm the biodistribution of cy5.5 and CUR *ex vivo*, the cancer-bearing mice were euthanized, and their organs (liver, lung, spleen, kidney, heart, and muscle) and tumor tissue were dissected and scanned by the imaging system. The biodistribution of cy5.5 and CUR in each organ or tissue was determined by calculating the fluorescence intensity of cy5.5 and CUR per weight of each organ or tissue scanned at 6, 24 and 48 hours.

### Assessment of* in vivo* anti-tumor effect

The experimental groups were as follows: tumor control, free CUR solution-, and PLL-DOCA-MPEG-cy5.5/CUR NP-treated groups. This study was performed in mice with a tumor volume of 200–400 mm^3^. CUR solution (0.1 mg/kg in 100 μL of water for injection) and PLL-DOCA-MPEG-cy5.5/CUR NPs (2.66 mg/kg in 100 μL of water for injection) were administered every third day via the lateral tail vein of Hep3B cancer-bearing mice (*n* = 7) for 27 days. The tumor volumes and body weights were measured at predetermined time intervals (0, 3, 6, 9, 12, 15, 18, 21, 24, 27, and 30 days). The tumor volume was calculated by the following formula: *V* = 0.5 × longest diameter × (shortest diameter)^2^. At day 30, liver and tumor tissues from each group were dissected for hematoxylin and eosin (H&E). The tissues were fixed in 10% neutral formaldehyde solution overnight. The fixed tissues were embedded in paraffin, and sectioned at 3 μm. The histological sections were classically stained with H&E. The stained slides were visualized by fluorescence microscopy (AX 70, TR-62A02, Olympus, Tokyo, Japan).

### Statistical analysis

All quantitative data were expressed as the mean ± standard deviation. Statistical analysis was performed with one-way analysis of variance (ANOVA) using SPSS software (SPSS Inc., Chicago, IL). A value of *p < .05 was considered statistically significant. The number of mice was calculated by MedCalc Statistical Software (MedCalc Software bvba, Ostend, Belgium) using Cronbach’s alpha (*p* = .05) and power (1-beta = 0.8).

### Institutional review board

The animal experimental protocols were approved by the Institutional Animal Care and Use Committee (IACUC) of Yeouido St. Mary’s Hospital of The Catholic University of Korea (no. YEO20153402FA).

### Results and discussion

#### ^1^H analysis of PLL-DOCA-MPEG-cy5.5 polymer

The conjugation of DOCA, MPEG-COOH, and cy5.5 to PLL chain was confirmed by ^1^H NMR spectroscopy using DMSO-*_d6_*(the upper spectrum of Figure 2(B)). DOCA, MPEG-COOH, and cy5.5 allow for conjugation with PLL, because their carboxylic groups can form amide bond with the amine group of the cationic polymer through condensation reaction (Park et al., 2009). DMT-MM was used as a condensation agent for amide bond formation due to its convenient one-step procedure (Kuishima et al., 2002). In the ^1^H NMR spectrum of PLL-DOCA-MPEG-cy5.5, characteristic peaks were observed at 3.24 ppm and 3.29–3.87 ppm for MPEG-COOH, respectively. The weak peaks at 7.0–8.0 ppm, and the singlets at 2.75 ppm and 4.15 ppm were assigned to cy5.5 and PLL, respectively. The multiplets at 1.05–1.92 ppm were assigned to the overlap of PLL and DOCA. These results indicated the successful conjugation of DOCA, MPEG-COOH, and cy5.5 to PLL. The degree of substitution (DS) of DOCA, MPEG-COOH, and cy5.5 was calculated by comparison of the integration ratio between their characteristic peaks and 2.75 ppm of PLL, and the DS was 13.3%, 6.7%, and 10.0%, respectively. As will be explained in detail in the next section, the micelle formation of PLL-DOCA-MPEG-cy5.5 was confirmed by comparing the ^1^H NMR data examined using D_2_O and DMSO-*_d6_*, respectively (Figure 2(B)). Some researchers have found that the hydrophobic moieties of amphiphilic polymers are self-assembled in water, resulting in the formation of particles or micelles (Park et al., 2009; Gou et al., 2011; Hu et al., 2017). The evident peaks corresponding to DOCA were observed at 0.60 ppm and 0.84 ppm in the spectrum using DMSO-*_d6_*(the upper spectrum of Figure 2(B)), whereas the peaks disappeared in the spectrum using D_2_O (the bottom spectrum of Figure 2(B)). Besides, PLL-DOCA-MPEG-cy5.5 polymer solution was clearly dissolved in DMSO-*_d6_* and D_2_O, respectively (Figure 2(B)). These results demonstrated that PLL-DOCA-MPEG-cy5.5 polymer formed micelles in water due to the self-assembly of DOCA.

### Determination of CMC and *in vitro* cytotoxicity of PLL-DOCA-MPEG-cy5.5 NPs

The conjugation of hydrophobic DOCA to PLL endows amphiphilic property that facilitates core–shell micelles formed by self-assembly in aqueous milieu. The CMC measurement of amphiphilic polymers using pyrene fluorescence spectroscopy is one of widely used methods for investigating the formation of their core–shell micelles (Wilhelm et al., 1991). The CMC was measured by plotting *I*_338_/*I*_332_ using the changes in fluorescent intensity at *I*_332_ (pyrene in a hydrophilic environment) and *I*_338_ (pyrene in a hydrophobic environment) (Wilhelm et al., 1991). The plot of *I*_338_/*I*_332_ of PLL-DOCA-MPEG-cy5.5 solution was examined as a function of concentration, resulting in a 0.69 × 10^−3 ^mg/mL of CMC (Figure S1(A)). PLL-DOCA-MPEG-cy5.5 dispersed in aqueous solution can self-assemble above the CMC through amphiphilic characteristics between PLL and DOCA. Even though the benefit of using nanoparticles as a DDS for cancer therapy is obvious, the nanocarriers have to be biocompatible, biodegradable and less immunogenic to obtain maximum therapeutic benefit. In this regard, the *in vitro* cytotoxicity of PLL-DOCA-MPEG-cy5.5 NP was evaluated. The cytotoxicity of Hep3B cultured with various concentrations of PLL-DOCA-MPEG-cy5.5 NPs *in vitro* was examined by CCK-8 assay (Figure S1(B)). For 72 hours of incubation, the NPs showed no serious cytotoxicity at any concentration tested (Figure S1(B)).

### Characterization of PLL-DOCA-MPEG-cy5.5/CUR NPs

**Figure 3.**
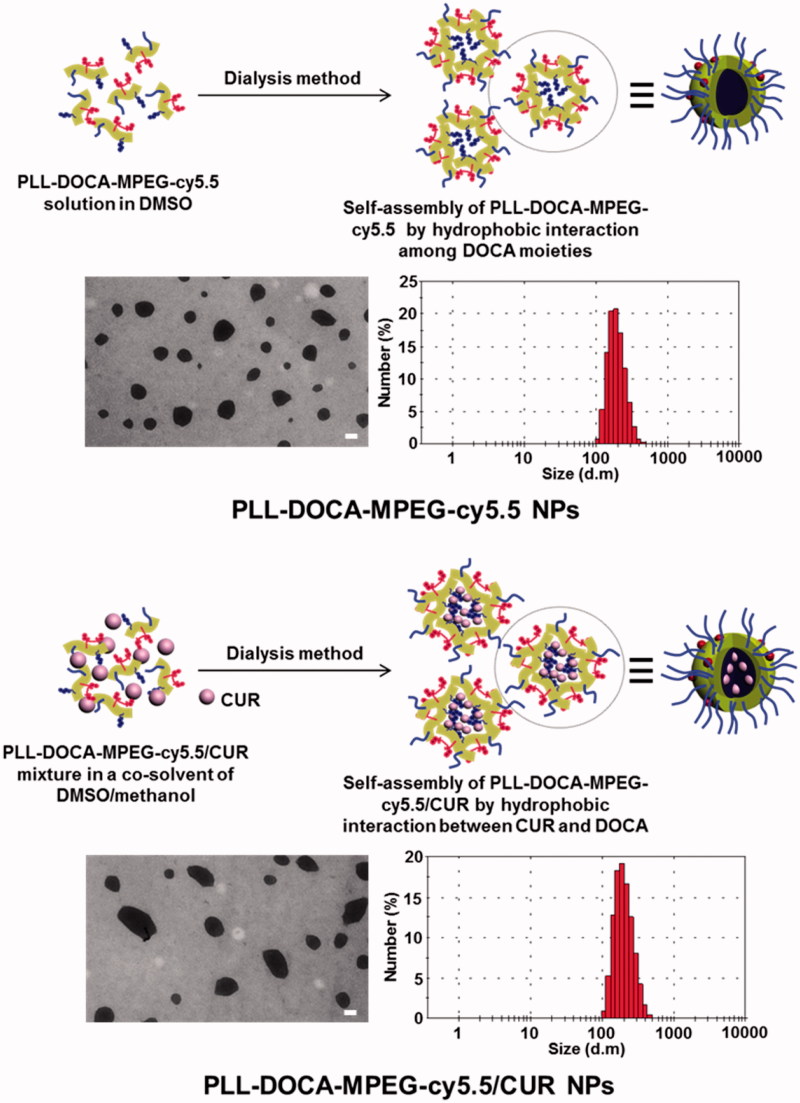
TEM images and size distributions of PLL-DOCA-MPEG-cy5.5 and PLL-DOCA-MPEG-cy5.5/CUR NPs. The scale bar (black line) of TEM images is 100 nm.

The morphology and size distribution of PLL-DOCA-MPEG-cy5.5/CUR NPs were analyzed by TEM and DLS as shown in Figure 3, respectively. Table S1 summarizes the EE and LC of the NPs on CUR as well as the average size, PDI, and zeta potential measured by DLS. Two kinds of NPs, PLL-DOCA-MPEG-cy5.5, and PLL-DOCA-MPEG-cy5.5/CUR had spherical morphologies with narrow size distribution (Figure 3) and positive zeta potential (Table S1). Compared with PLL-DOCA-cy5.5 NPs (229 ± 10.3 nm), a slight increase in the size distribution was observed for PLL-DOCA-MPEG-cy5.5/CUR NPs (246 ± 5.8 nm). This was attributed to the encapsulation of CUR in PLL-DOCA-MPEG-cy5.5 NPs by hydrophobic interaction between CUR and DOCA to increase the core volume of NPs. Hydrophobic substances, such as hydrocarbon chains, cholesterol derivatives, and so on, are generally used as core parts of amphiphilic polymer-based NPs that induce the loading of drugs of poor water solubility through hydrophobic interaction (Trivedi & Kompella, 2010). Among these, DOCA might be the best candidate for the hydrophobic core part of PLL, because the introduction of hydrophobic segments into PLL is simply achieved by grafting carboxyl groups to free amine groups of PLL. In addition, DOCA is a main component of bile acid that solubilizes the fats for absorption into the intestine and has a hydrophobic cyclopentenophenanthrene ring structure in its molecule; therefore, DOCA-grafted amphiphilic polymers in aqueous environments are known to form nano-sized micelles with a unique core–shell structure (Yang et al., 2014; Faustino et al., 2016). Accordingly, DOCA was chosen and conjugated to the PLL backbone for the formation of amphiphilic PLL-based NP. The DOCA core has a strong hydrophobic interaction with CUR, resulting in improved loading efficiency. As listed in Table S1, PLL-DOCA-MPEG-cy5.5 NPs exhibited 78.53 ± 2.31% of EE and 13.12 ± 1.29% of LC on CUR, respectively (Table S1).

### Size distributions and zeta potentials of charge reversal PLL-DOCA-MPEG-cy5.5/CUR NPs, and *in vitro* release behavior of CUR

Tumor has lower extracellular pH than normal tissues (Gao et al., 2010). It is primarily due to the high metabolic rate of tumor but insufficient removal of by-products (Gao et al., 2010). Therefore, NPs for intravenous (IV) administration circulate various pH circumstances from veins to the tumor via EPR as shown in Figure 1(ii). PLL-DOCA-MPEG-cy5.5 NPs, as cationic NPs, become positively charged and swell due to protonation at low pH. In addition, the swelling of PLL that affects the release of CUR depends entirely on the level of protonation. Therefore, it is important to determine the changes in size and charge of NPs under minimum three pH conditions as depicted in Figure 1(ii–iii); IV blood stream (pH 7.3–7.4), tumor extracellular environment (pH 6.8) and endosomal environment of (pH 5.5), respectively (Gao et al., 2010). Figure S2 shows the size distributions and zeta potentials of PLL-DOCA-MPEG-cy5.5/CUR NPs (Figure S2(A)) and the release behavior of CUR (Figure S2(B)) as a function of pH (5.5, 6.8 and 7.4). The changes in morphology and the drug release behavior might be influenced by the pH change of cationic polymers as reported by some studies (Patra & leem, 2013; Mouslman et al., 2015). As pH decrease, PLL that forms the shell of the NPs become protonated. Once protonated, the entangled PLL chains under neutral pH started to swell due to cationic repulsive forces between chains resulting in the increase in the volume of NPs (Xu et al., 2006). Considering that the average size of NPs increases more than 2.5 fold at pH 5.5, the release of CUR increases dramatically, resulting in initial burst release.

### Cellular uptake and anti-tumor activity of PLL-DOCA-MPEG-cy5.5/CUR NPs *in vitro*

**Figure 4.**
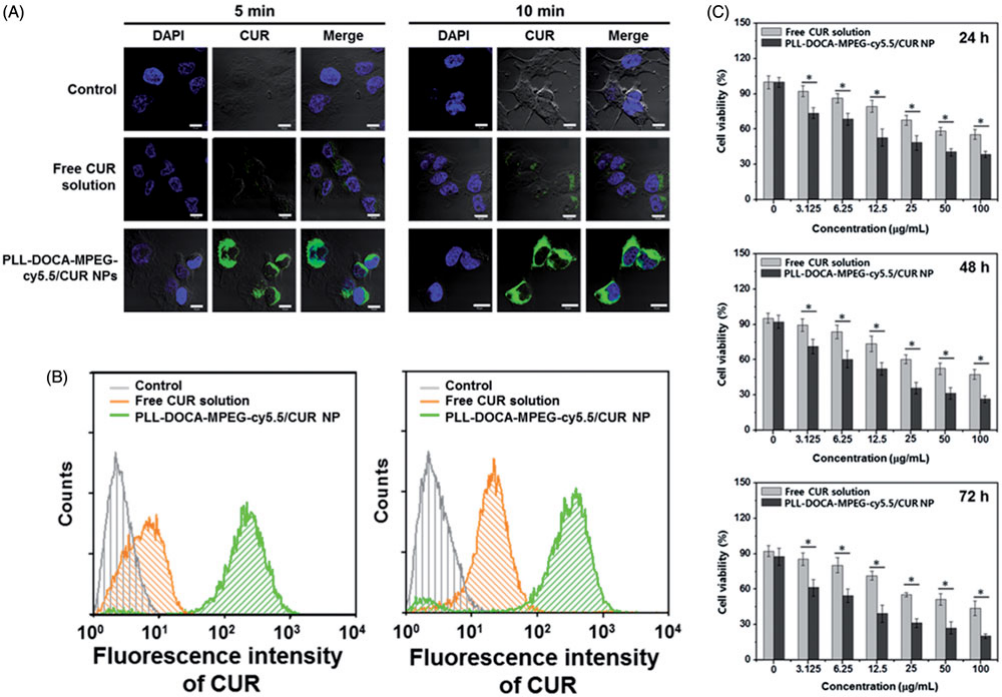
(A) CLSM images and (B) flow cytometry data on cellular uptake of free CUR solution and PLL-DOCA-MPEG-cy5.5/CUR NPs into Hep3B cells at 5 and 10 minutes of treatments (corresponding to 0.09 μM CUR) *in vitro*. (C) *In vitro* cell viability of Hep3B cells cultured on PLL-DOCA-MPEG-cy5.5 NPs, determined by measuring the fluorescence spectra of pyrene in solution. The images were observed by CLSM. Blue and green colors indicate DAPI and CUR, respectively. The scale bar (white line) of each image is 10 μm. Flow cytometry was employed to examine the association of free CUR or PLL-DOCA-MPEG-cy5.5/CUR with Hep3B cells. The *in vitro* cell viability (%) was determined by CCK-8 assay at various concentrations of NPs (0–100 μg/mL) after 24, 48 and 72 hours of incubation. Error bars represent mean ± SD (*n* = 3); the *in vitro* cell viability test was repeated three times (*p < .05 compared with free CUR solution groups).

The basic strategy of the pH sensitive DDS is to attract and adsorb NPs to the negatively charged cell membrane to lead electrostatically absorptive endocytosis. CLSM and flow cytometry can be very useful tools to visualize and quantify this kind of endocytosis, respectively (Kumari et al., 2017). Figure 4(A) shows the confocal images of Hep3B cells treated with free CUR solution and PLL-DOCA-MPEG-cy5.5/CUR NPs for 5 and 10 minutes *in vitro*, respectively, compared with control. No fluorescence was observed in the cells of control. Free CUR solution whose transportation within the cell depends solely on passive diffusion was localized intracellularly. Meanwhile, rapid accumulation of CUR within the cytosol was found in Hep3B cells treated with PLL-DOCA-MPEG-cy5.5/CUR NPs. The flow cytometry analysis showed that the accumulation of CUR in Hep3B cells treated with PLL-DOCA-MPEG-cy5.5/CUR NPs, in a quantitative respect, increased beyond a digit compared to cells treated solely with free CUR (Figure 4(B)). As shown in Figure 1, tumor extracellular environment has extracellular acidification to pH 6.8 because the high level of lactate produced by tumor cells contributes to the acidic environment (Park et al., 2001; Hayden et al., 2012). Consequently, the amine group of PLL is changed to NH_3_^+^ and causes the cationic properties of PLL-DOCA-MPEG-cy5.5/CUR NPs, allowing for a favorable interaction between the NPs and the negative cell membrane (Park et al., 2001; Hayden et al., 2012). It was therefore reasonable to assume that cationic charged PLL-DOCA-MPEG-cy5.5/CUR NPs have higher cellular uptake rate than free CUR. So, CUR delivered in tumor cells by PLL-DOCA-MPEG-cy5.5/CUR NPs may induce enhancing anti-tumor activity. To verify the anti-tumor activity of Hep3B cells on PLL-DOCA-MPEG-cy5.5/CUR NPs, the cell viability using a CCK-8 assay was conducted and compared with those on control and free CUR solution (Figure 4(C)). As expected, PLL-DOCA-MPEG-cy5.5/CUR NPs showed superior anti-cancer activity compared with free CUR over every dose concentration throughout the culture periods.

### *In vivo *and* ex vivo* NIRF imaging, and *in vivo* anti-tumor effect

**Figure 5.**
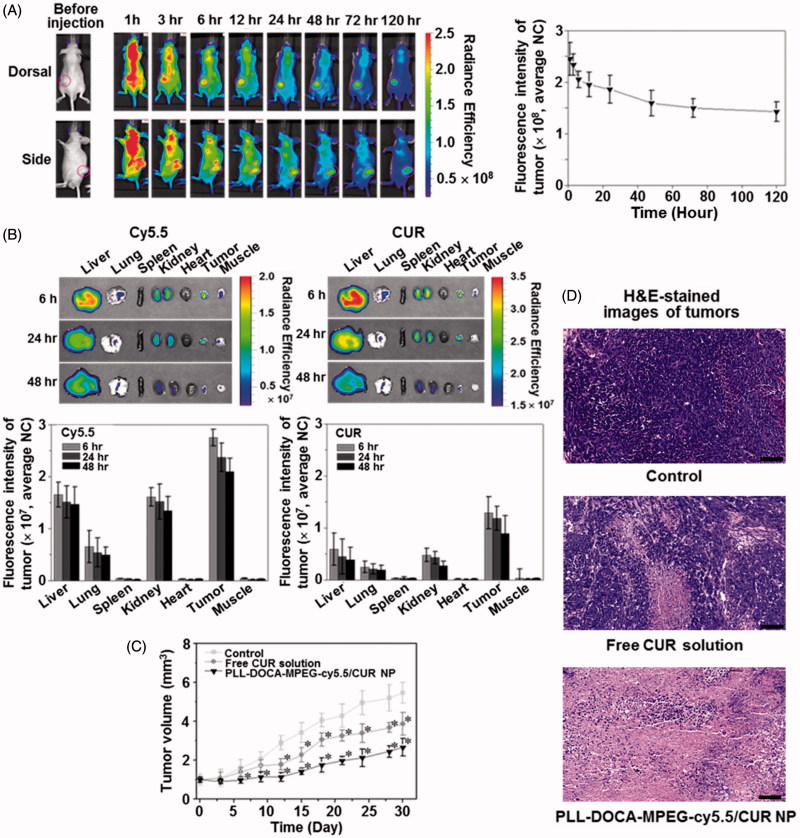
(A) *In vivo* whole body NIRF images of Hep3B cancer-bearing mouse models. PLL-DOCA-MPEG-cy5.5/CUR NPs were injected via the lateral tail vein. Real-time images were taken at 0, 1, 3, 6, 12, 24, and 48 hours. The tumor tissues were marked with open pink circles. The whole body images were scanned on the dorsal and side. Fluorescence intensity profiles of cy5.5 and CUR in cancer region over time, which were determined by calculating the fluorescence intensity of cy5.5 and CUR per weight of cancer. Error bars represent mean ± SD (*n* = 3). (B) *Ex vivo* NIRF images and fluorescence intensities of cy5.5 and CUR on liver, lung, spleen, kidney, heart, cancer and muscle, measured at 6, 24, and 48 hours after the IV injection. The fluorescence intensities were determined by calculating the fluorescence intensity of cy5.5 and CUR per weight of each organ or tissue. (C) Tumor volume (mm^3^) and of mice treated with free CUR solution and PLL-DOCA-MPEG-cy5.5/CUR NPs or control on day 30. Free CUR solution and PLL-DOCA-MPEG-cy5.5/CUR NPs were intravenously injected via the lateral tail vein on days 3, 6, 9, 12, 15, 18, 21, and 27. Error bars represent mean ± SD (*n* = 5); the measurement of the cancer volume was repeated three times (*p < .05 compared with control). (D) H&E-stained images of untreated tumor tissue (control), and dissected tumor tissues treated with free CUR solution and PLL-DOCA-MPEG-cy5.5/CUR NPs on day 30. The black scale bar is 100 μm.

An IV administration system is one of the most efficient drug delivery methods because the onset of drug action is rapid following the injection, and smaller doses are required than with other routes of administration (Jain, 2008). Therefore, the IV DDS could be a first option for drugs with poor solubility. The particle size for the IV DDS is very important because the bio-distribution of particles to various organs is dependent upon their sizes (Jain, 2008). For instances, particles larger than 7 μm are trapped in the lungs, those between 0.1 and 7 μm are taken by the liver and spleen, and those smaller than 0.1 μm can be accumulated in the bone marrow (Jain, 2008). This is very useful information to target the organ in the IV DDS; however, there exist miscellaneous have to be considered in materials aspects and/or metabolic profiles. In the case of DDS using PLL, the first thing to be considered is the surface charge of PLL. In previous reports, surface cationic charges of PLL caused aggregation with plasma proteins (Ziady et al., 1999; Ward et al., 2001). Although they used the spherical PLL-based NPs with ranging from 100 nm to 500 nm, the aggregation increased the size of NPs several folds larger which is enough to prevent extravasation as well as to create several embolisms in vasculatures or in tissue organs. In addition, the abnormal aggregates in tissue organs may trigger continuous inflammatory reactions with the chronic cells; the Kupffer cells of liver or the macrophages of spleen (Ziady et al., 1999; Ward et al., 2001). Therefore, PEGylation is necessary in order to reduce serum inhibition and to evade the recognition and subsequent uptake by the mononuclear phagocyte system, thus to have a circulation time in blood stream for stable drug delivery to tumor via EPR effect (Figure 1(ii)) (Gref et al., 1994). Figure 5(A) shows the NIRF imaging of PLL-DOCA-MPEG-cy5.5/CUR NPs on whole body *in vivo*, monitored over 120 hours. The NPs appeared to be distributed to whole body and gradually excreted from normal organs for up to 120 hours after circulation. There were no signs of abnormal aggregation of NPs in vasculature and tissue organs that strongly demonstrated the effect of PEGylation of NPs. At 120 hours, the NPs were mainly detected in the tumor, and most of them in normal organs had been excreted.

In order to investigate the delivery of CUR to the individual organs, the *ex vivo* biodistribution of PLL-DOCA-MPEG-cy5.5/CUR NPs in liver, lung, spleen, kidney, heart, tumor, and muscle was examined by recording the fluorescence intensity of cy5.5 and CUR over 48 hours (Figure 5(B)). At each time point, the tumor showed stronger fluorescence intensity of cy5.5 and CUR than the normal organs (Figure 5(B)). These results strongly proved the enhanced permeability retention (EPR) effect of PLL-DOCA-MPEG-cy5.5/CUR NPs. In addition, the most important phenomenon to be discussed here is the cellular uptake of PLL-DOCA-MPEG-cy5.5/CUR NPs via electrostatic absorption, because PEG chains are likely to slow down the nanoparticle cellular uptake due to steric repulsion (De Jaeghere et al., 2000). This can be explained by the pH sensitivity of PLL-DOCA-MPEG-cy5.5/CUR NP. As discussed in Figure S2, under tumor extracellular environment protonated PLL chains become hydrophilic corona that surround the core with PEG. The positively charged PLL corona may result in rapid cellular internalization (Figure 1) (Blau et al., 2000). The continuous protonation of NPs in endosome and lysosome (pH 5.5) produce PLL become swell up result in dissolution of NPs, in turn, lead to the lysosomal rupture due an osmotic swelling, and finally, to a rapid drug release into the cytoplasm as represented in Figure 1 (Xu et al., 2006). Unlike the PLL-based NPs, some amphiphilic polymers, such as PEG-polyester, control the release of CUR by hydrolysis. The hydrolysis causes a slower release of CUR in cancer cells than PLL-DOCA-MPEG-cy5.5/CUR NP (Jin et al., 2017); therefore, polyester-based NPs may result in lower anti-tumor activity.

The above-mentioned biodistribution and pH responsive characteristics of PLL-DOCA-MPEG-cy5.5/CUR NP seemed to be directly reflected *in vivo* anti-tumor activity. Throughout 30 days, PLL-DOCA-MPEG-cy5.5/CUR NP showed the lowest growth rate of tumor volume in the groups tested, indicating remarkable anti-tumor activity (Figure 5(C)). The anti-tumor activity was further confirmed by histological analysis of tumor tissues (Figure 5(D)). Compared with control, a part of necrotic area was found in the tumor treated with CUR solution, whereas PLL-DOCA-MPEG-cy5.5/CUR NP-treated tumor exhibited a wide range of necrosis over the whole area (Figure 5(D)). The results in Figure 5 provided the strong evidence that PLL-DOCA-MPEG-cy5.5/CUR NP had the long circulation time in blood vessels for the enhanced EPR effect and that CUR was continuously delivered via electrostatic adsorption between PLL and cell membrane.

### Systemic toxicity assay *in vivo*

**Figure 6.**
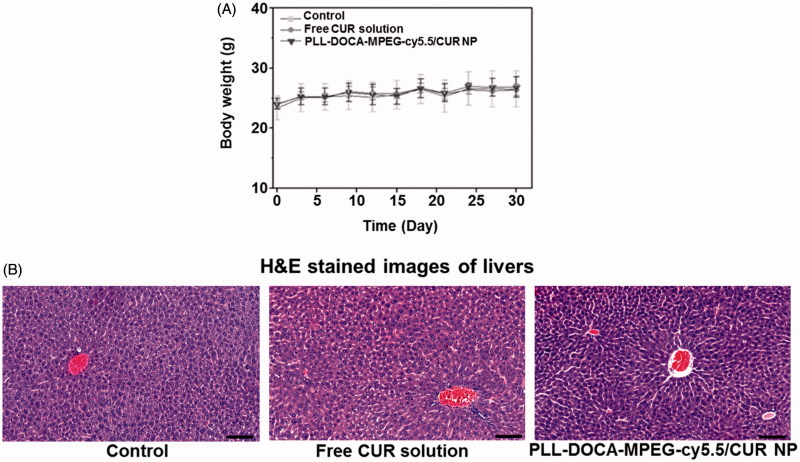
(A) Body weights of normal mice (control), and mice treated with free CUR solution and PLL-DOCA-MPEG-cy5.5/CUR NPs. (B) Histological assays of untreated liver tissue (control), and dissected liver tissues treated with CUR solution and PLL-DOCA-MPEG-cy5.5/CUR NPs on day 30. The black scale bar is 100 μm.

The systemic toxicity of PLL-DOCA-cMPEG-cy5.5/CUR NPs was evaluated in terms of the changes in body weight and the liver toxicity of mice treated with the NPs (Figure 6). As shown in Figure 6(A), in common with control and free CUR-treated groups, little change in the body weight was observed in the NP-treated group, suggesting no serious toxicity. Figure 6(B) shows the cross-sections of liver of control and NPs treated mice. All images represent the basic structure of liver lobule; the sinusoids are spread radially around the central vein, and hepatocytes are present between perisinusoidal spaces. There are no signs of inflammation, fibrosis, and cirrhosis.

## Conclusions

In this study, we prepared PLL-based NPs composed of PLL, DOCA, MPEG, and cy5.5, PLL-DOCA-MPEG-cy5.5/CUR NPs using a dialysis method in aqueous solution for the cancer theranosis. PEGylation with MPEG to NPs was turn out to be very effective to have a prolonged blood circulation time, in turn increase the EPR effect. PLL, shell of NPs, sensitively responded to low pH to increase the electrostatically absorptive endocytosis. In addition, the incorporation of Cy5.5 to NPs provided the successful biodistribution images *in vivo* and *ex vivo*. Consequently, PLL-DOCA-MPEG-cy5.5/CUR NPs showed superior ability for cancer imaging and therapy.

## Supplementary Material

IDRD_Chun_et_al_Supplemental_Content.pptx
