# Supplementary figures and images for: Preparation of poly-l-lysine-based nanoparticles with pH-sensitive release of curcumin for targeted imaging and therapy of liver cancer in vitro and in vivo
Source: Drug Deliv. 2018 Apr 16;25(1):950–60. doi: 10.1080/10717544.2018.1461957 (PMC6058614; doi:10.1080/10717544.2018.1461957)

## Slide 1
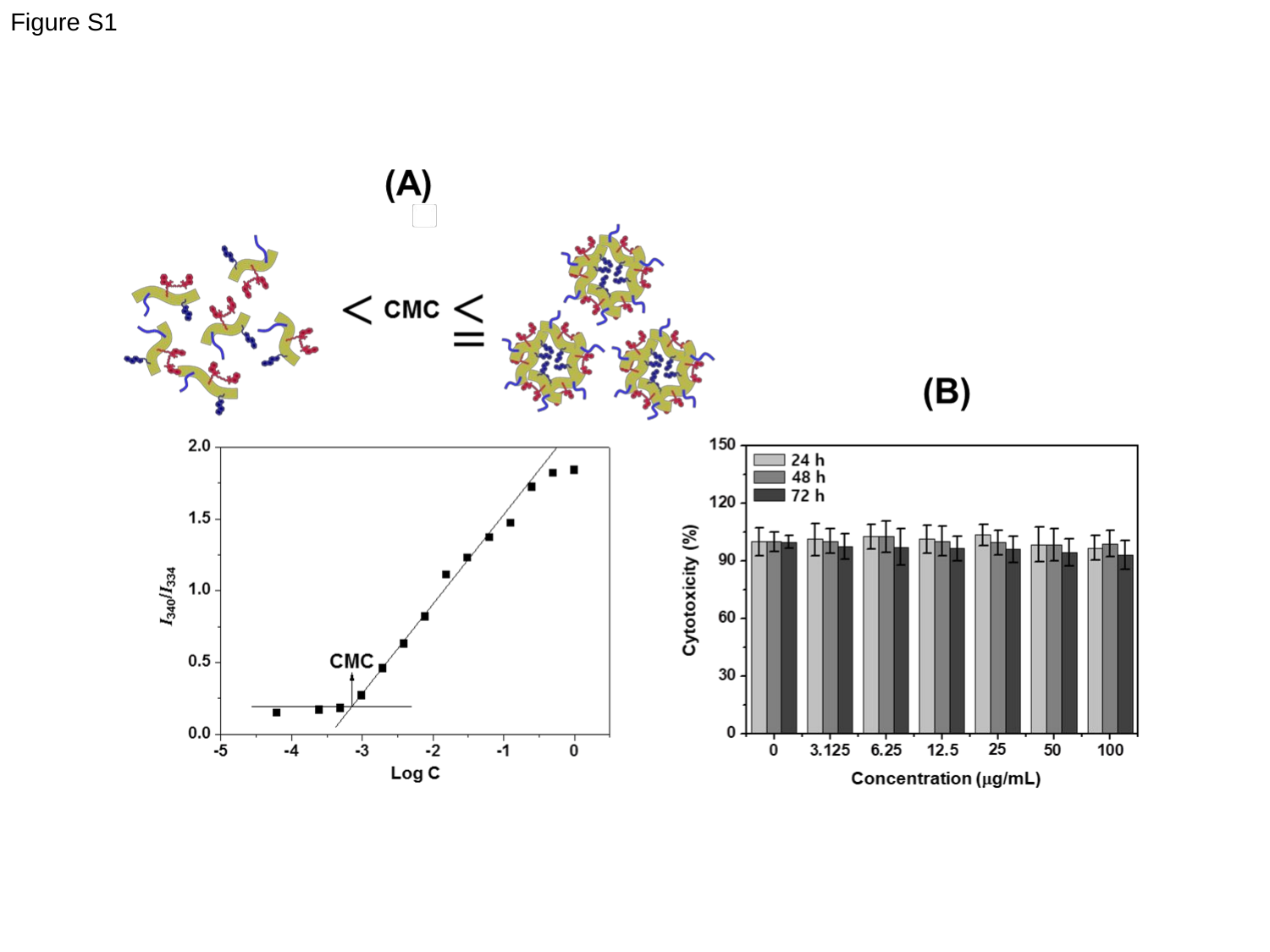

Figure S1

## Slide 2
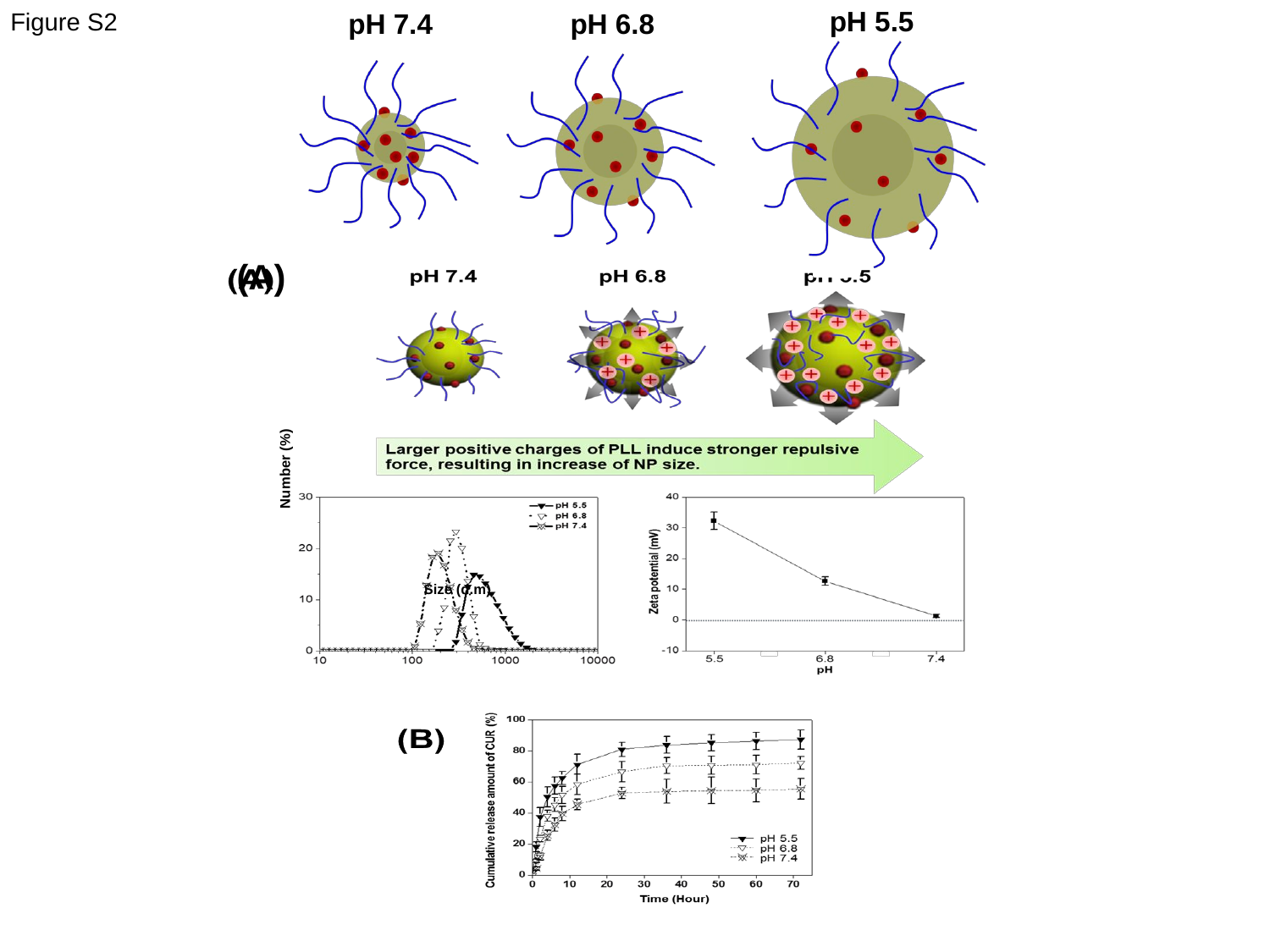

Figure S2
pH 7.4
pH 6.8
pH 5.5
(A)
Number (%)
Size (d.m)

Supplement: IDRD_Chun_et_al_Supplemental_Content.pptx [file IDRD_A_1461957_SM0016.pptx]
